# Medical Needling: Effect on Moisture and Transepidermal Water Loss of Mature Hypertrophic Burn Scars

**DOI:** 10.7759/cureus.2365

**Published:** 2018-03-26

**Authors:** Kay-Hendrik Busch, Antigona Aliu, Nicole Walezko, Matthias Aust

**Affiliations:** 1 Waldkrankenhaus Bonn, Johanniter Kliniken Bonn; 2 Medical Faculty, Heinrich-Heine-University; 3 Anästhesiologie, Marienhospital Bonn; 4 Plastic Surgery, Waldkrankenhaus Bonn

**Keywords:** minimal invasive technique, low-risk therapy, hydration, transepidermal water loss, moisture, barrier function

## Abstract

Background: Burn scars remain a serious psychological and physiological problem for affected people. Clinical studies and scientific research have already shown that medical needling improves the scar quality in terms of skin elasticity and erythema. At the same time, patients are confronted with a low-risk therapy and face comparatively less postoperative complications.

Objective: The goal of our study was to examine the influence of medical needling on the skin moisture and transepidermal water loss (TEWL) of hypertrophic dry scars. Therefore, 20 patients, of an average age of 34.63 years, with deep second- and third-degree burn scars have been treated.

Methods: Medical needling is performed using a roller covered with needles of 3-mm length. The needling device is rolled over the scar in three directions: vertically, horizontally, and diagonally in order to create as many puncture channels as possible. The puncturing leads to multiple micro-wounds and intradermal bleeding, which evokes the post-needling regeneration cascade. The patients were followed up for 12 months postoperatively. The results have been evaluated by means of objective as well as subjective measurement methods.

Results: The objective measures show that medical needling influences epidermal thickness and improves the epidermal barrier function at a molecular level. Outcomes are marked by a measurable increase in skin moisture and a reduction in TEWL.

Conclusion: Medical needling seems to be a promising approach for the treatment of mature hypertrophic burn scars with a focus on skin moisture and TEWL.

## Introduction

Burns are the fourth leading cause of injuries worldwide, resulting in different types of scars [1]. There are approximately 11 million affected people each year who suffer from burns and require medical treatment. Due to the progression of modern medicine and the development of innovative methods, the mortality rate has significantly decreased over the past decades. Burn victims are often confronted with dysfunctional and aesthetic deficits in their daily life. Typical consequences involve psychological stress and social isolation. For this reason, the demand for less invasive but effective medical treatments is steadily growing. In a full thickness burn, disturbed and delayed wound-healing processes frequently cause the formation of pathological and noticeable scars. Widespread tissue defects and the severe destructive damages of dermal and epidermal structures do often result from secondary wound healing. Consequently, the formation of pathological scars reveals the typical characteristics of optical impairment, pain, and itching [2-3]. A major problem is the development of hypertrophic scars with a prevalence of 67%. The current difficulty resides in skin dryness and the insufficient water content of the epidermis [4-5]. Dehydrated scar conditions are the main cause of pruritus, which is closely related to the impaired epidermal structure of the scarring [6]. As scar tissue has fewer skin appendages, such as sebaceous and sweat glands, the skin produces less moisturizing secretions, which leads to skin dryness. Another causative factor for pruritic scars is a thinner epidermis of the scar with a parallelly orientated collagen structure. This collagen matrix can have a negative impact on the epidermal barrier function [7-8].

Severe burns hold the risk of an incomplete re-epithelialization of the epidermis due to a lack of skin appendages as a stem cell reservoir. Thus, a less functional epidermis enhances transepidermal water loss (TEWL) and a reduction in moisture. For this reason, patients suffer from itching and painful scars followed by reddened and vulnerable skin. An impaired epidermis with deficient protective features reacts more sensitively to external factors and is susceptible to penetration by pathogens. In this context, skin moisture and TEWL are important indicators in order to measure the efficiency of the skin barrier [9]. This study is aimed at examining whether medical needling improves the moisture content of the skin, which is closely interlinked with TEWL. Therefore, we performed measurements with the Corneometer (Courage-Khazaka Electronic, Cologne, Germany) and the Tewameter (Courage-Khazaka Electronic, Cologne, Germany), implying the moisture and TEWL index, in order to determine significant changes. The current medical knowledge offers a diversity of possibilities for the treatment of dry and itching scars with different therapeutic approaches and success. The very common practice offers conservative treatments for scars with deficits in moisture and TEWL, including silicone patches or moisturizing creams as established therapy methods. Provided that they are used permanently over a longer period of time, both can improve the scar condition temporarily [10]. Regarding the water content, the fluid silicone gel preserves the moisture of the skin but does not affect the capacity of the skin to produce moisture [11-13]. The constant application of oily creams also keeps the skin moist and smooth but does not improve the scar quality itself. On the contrary, TEWL is less affected, as the epidermal barrier function is not influenced by the silicone. Consequently, these methods are rather suitable for short-term usage and regarding TEWL, there are less satisfying outcomes. For this reason, these methods have a preventative character and are rather suited as short-term solutions or for temporal improvement [14]. Ablative treatments, such as laser resurfacing or dermabrasion, destroy epidermal structures and, thus, hold the risk for degradation of the scar condition [15-17]. Hence, the epidermis is thinner, which causes water-related deficits of the skin. Moreover, the affected area is more sensitive to bacterial infections, leading to an inflammatory response, which, in turn, requires additional treatment. Compared to that, percutaneous collagen induction or medical needling overcomes the shortcomings of conventional treatments with a focus on sustainability. Therapy outcomes are up-to-date and have been already examined in several previous studies. The procedure of medical needling can be safely repeated and used in regions where conventional treatments have limited usage. The minimally invasive and non-ablative method does not destroy the epidermis but rather promotes the formation of physiological collagen instead of scar collagen. The stimulation of a natural wound healing cascade and the expression of specific growth factors initiate an ideal regeneration of the skin in terms of a dermal reorganization. Subjective improvements of the scar situation have been already proved by both clinical and scientific data. The aim of this study is to evaluate the effect of medical needling on skin moisture and TEWL by means of objective and quantitative measurement methods.

## Materials and methods

Study design

This study is a prospective, randomized, controlled within-subject comparison. The subject’s hypertrophic scar areas were divided into two subareas for which treatment was randomly allocated as (1) medical needling (positive control) and (2) no treatment (negative control). In order to exclude a possible autonomous healing of the examined scars, the untreated scars of the patients were involved (control group). The patient cohort was not preselected with reference to scar quality before the study. An evaluation of therapeutic outcome regarding moisture and TEWL has been performed at the baseline and within a three, six, and 12-month postoperative examination of the treated and untreated scars as well as the healthy reference of each participant. Preoperative examinations of the scar were followed by postoperative measurements after each treatment with medical needling. Postoperative results after one year were involved in the final statistical analysis in order to guarantee uniform assessment quality based on sustainable outcomes regardless of seasonal fluctuations and other external factors. Postneedling scar outcomes were assessed by both using photo documentation as well as the patient and observer scar assessment (POSAS). Measurements with the Corneometer offered reliable data about skin moisture. For the assessment of the TEWL in burn scars, it was aimed to investigate the reliability of the Tewameter.

Subject selection

Patients were required to have mature hypertrophic burn scars that have healed by secondary intent, were at least 10 cm^2^ in size, and were two years' post-accident. Scars, which are at least 2 years' old, have met the criteria for taking part in the study. Exclusion criteria were severe underlying diseases, skin lesions like infections, cancer, and pregnancy. Furthermore, involved scars were not treated in any additional form during the study.

Procedure

In Germany, medical needling is a licensed therapy for burn scars that is used frequently in the clinical practice. All pre- and postoperative examinations were performed in vivo. The entire procedure including general anesthesia and medical needling is executed in an operation theatre. According to the conditions of participation, all patients signed an informed consent form and declared their willingness to take part in the follow-up examination as well as in photo documentation. Informed consent was obtained from the parents of patients younger than 18 years of age. Preoperative management included the application of vitamin A, C, and E containing creams in order to create optimal conditions. The positive effect of this preoperative intervention has been already researched and described in the studies of Aust et al. [18].

Medical needling

**Figure 1:**
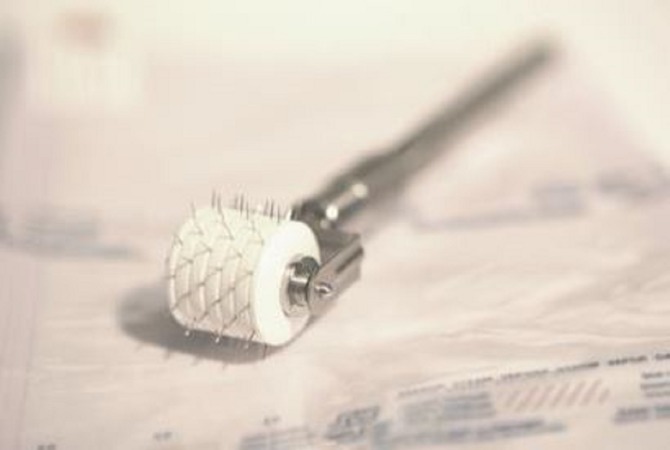
Roller device for medical needling

Medical needling is performed by rolling a device covered with 3-mm-long needles over the scar (Figure 1). The device needs to be rolled over the scar in three directions with constant pressure: vertically, horizontally, and diagonally. To prevent the wounds caused by shear forces it is important to keep the roller in a straight line. According to the extent of the scar, this procedure can take 30-60 minutes.

The multiple puncturing of the papillary dermis leads to thousands of micro-wounds, causing intradermal bleeding through the parenchymal canals. The penetration into the dermis disrupts the scar collagen that connects the dermis to the upper layers of the skin. Epidermal structures remain intact and skin cells are not repressed or destroyed within this procedure. The expression of specific growth factors and the release of structural proteins activate a natural wound-healing cascade. The scar is sufficiently needled when multiple and confluent ecchymoses develop and the skin is swollen. Medical needling does not produce open wounds and histological findings prove a re-epithelialization of the epidermis after 24 hours. For this reason, the postoperative complication rate is very low and post-interventional monitoring is not necessary. In order to maximize the outcome, the application of nourishing products is continued in the first 24 hours postoperatively, every three hours. The swelling and erythema of the treated area disappear after approximately four to seven days.

Assessment

Outcomes were assessed subjectively using POSAS and objectively using Corneometer CM 825 and the Tewameter for the in vivo examination of skin moisture and TEWL. POSAS is a patient and observer-administered scale that covers an array of scar characteristics and photos. In this study, we concentrate on the subjective evaluation of rigidity, itching, and thickness. Moreover, each scar was photographed before and after the treatment. The setting, the position of the subject, and light conditions were selected uniformly.

The practical implementation of the Corneometer is based on a capacitance measurement examining the water content of the stratum corneum of the epidermis. Two metal plates are isolated by dielectric devices functioning as a condenser. This is connected to a voltage source and stores electric charge when the electric current flow is adjusted. The capacitance represents the quantitative amount of charge, which depends on the dielectric properties of the stratum corneum relative to the degree of moisture penetration. Capacity constants are predefined for water (81) and the condenser (7). Hence, the amount of water in the measured skin alters proportionally to the capacity of the condenser, which gives the moisture index. Measurements were made three times in a row within 1 cm^2^ at the same skin location. They included each of the subjects‘ scars, treated and untreated, as well as healthy skin for comparison.

The TEWL measurements performed by the Tewameter are based on the principle of diffusion. The determination of water evaporation through the skin requires a practical device with ventilated chambers, which establishes a vapor pressure gradient. In terms of that, the device, containing two probes that are placed at a defined distance, determines the temperature and moisture of the affected area. Furthermore, it records the evaporated water from the skin surface and measures the partial pressure of water vapor. The proportional relation between the pressure drop and the evaporation rate gives the TEWL index. This method is less affected by disruptive external factors such as unstable pressure or temperature conditions. Measurements of 20 seconds, on average, were made for each of the subjects‘ scar, treated and untreated, as well as healthy skin for comparison.

Statistics

A statistical analysis was performed using the SPSS software (IBM, Armonk, New York, United States) and data collection was done using Microsoft Excel for Windows (Microsoft, Washington, United States). Due to the sample size of 20 participants, the "Wilcoxon signed rang text“ was used. Statistical significance was accepted at a level of p < 0.05.

## Results

Subjects

Twenty subjects, one Asian and 19 Caucasian, were involved in this study. The average age was 34.63 years, with a range from 6 to 60 years. The majority of injuries were due to barbecue accidents (31.6%) followed by scaldings with hot fluid (21.1%) and burns (21.1%). The diagnosis of thermal damage was given by 19 patients. One subject was the victim of an acid attack, which showed a similar damage profile as the thermal impairment. The average rate of the burned body surface was 32.42% and the average age of the scar at the time of the treatment was 11.42 years.

POSAS

The data of 19 patients who appeared at the follow-up examination are considered. The values from each subject‘s last visit (6-12 months) were used. There were no infections detected in any subject and all scars were 100% epithelialized. Subjective data based on POSAS points showed an improvement in the scar quality, with a significant tendency to normal skin. Each category was evaluated by the patient and the observer under standardized conditions. With this study, we were able to support previous studies regarding the effect of medical needling on itching [19, 20]. The parameter "pruritus“ was evaluated with a mean decrease of 73%, which is a significant improvement of the scar quality.

Patient ratings

**Figure 2:**
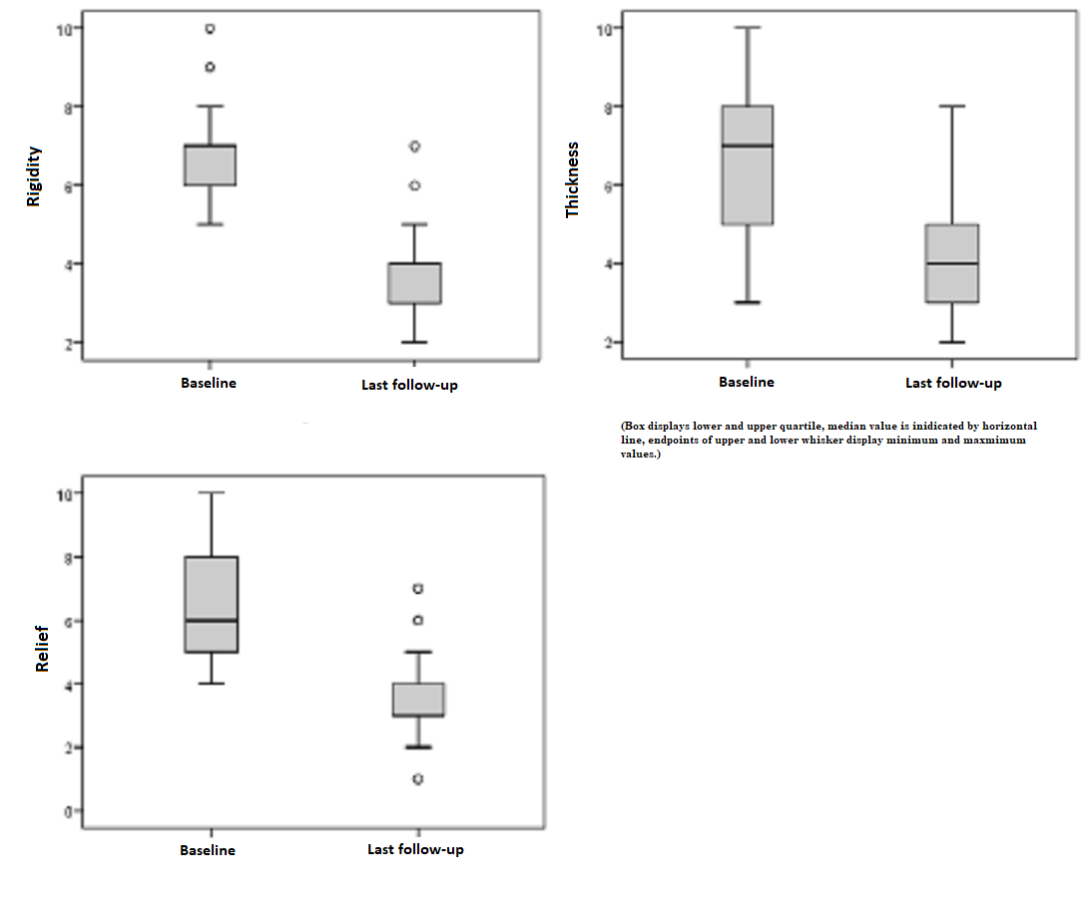
Patient ratings for "rigidity," "thickness," and "relief" preoperatively and at last follow-up Patient ratings: 1 = as normal skin, 10 = very different from normal skin

Subjects scored scar rigidity with a mean value of 6.74 points preoperatively, which decreased to 3.79 points postoperatively. The pre- and postoperative difference reveals a 51% reduction in the examined parameter and shows a tendency toward smooth and supple skin. Similar outcomes could be reliably determined considering the parameters "thickness" and "relief." The last-mentioned parameter shows an adjustment of 57% toward healthy skin conditions (Figure 2).

Observer ratings

**Figure 3:**
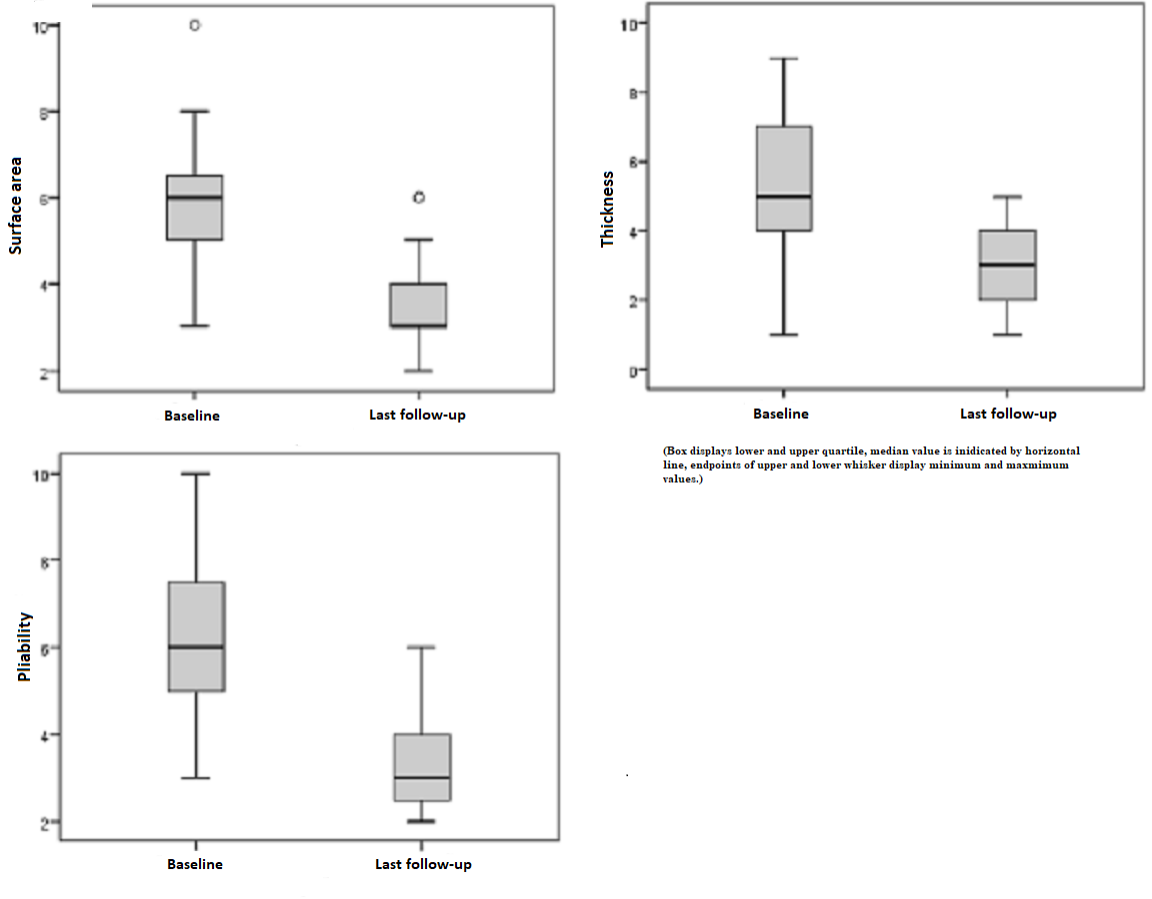
Observer ratings for "surface area," "thickness," and "pliability" preoperatively and at last follow-up Observer rating: 1 = as normal skin, 10 = very different from normal skin

Subjective data provided by the observer created a homogenous image: The surface area was preoperatively rated with a mean value of 5.84 points and postoperatively with 3.47 points at the last follow-up. For the skin area treated by medical needling, the pre- and postoperative difference means an adjustment of approximately 50% toward healthy reference conditions. This result also applies to the parameters "thickness" and "pliability" (Figure 3).

Photo documentation

**Figure 4:**
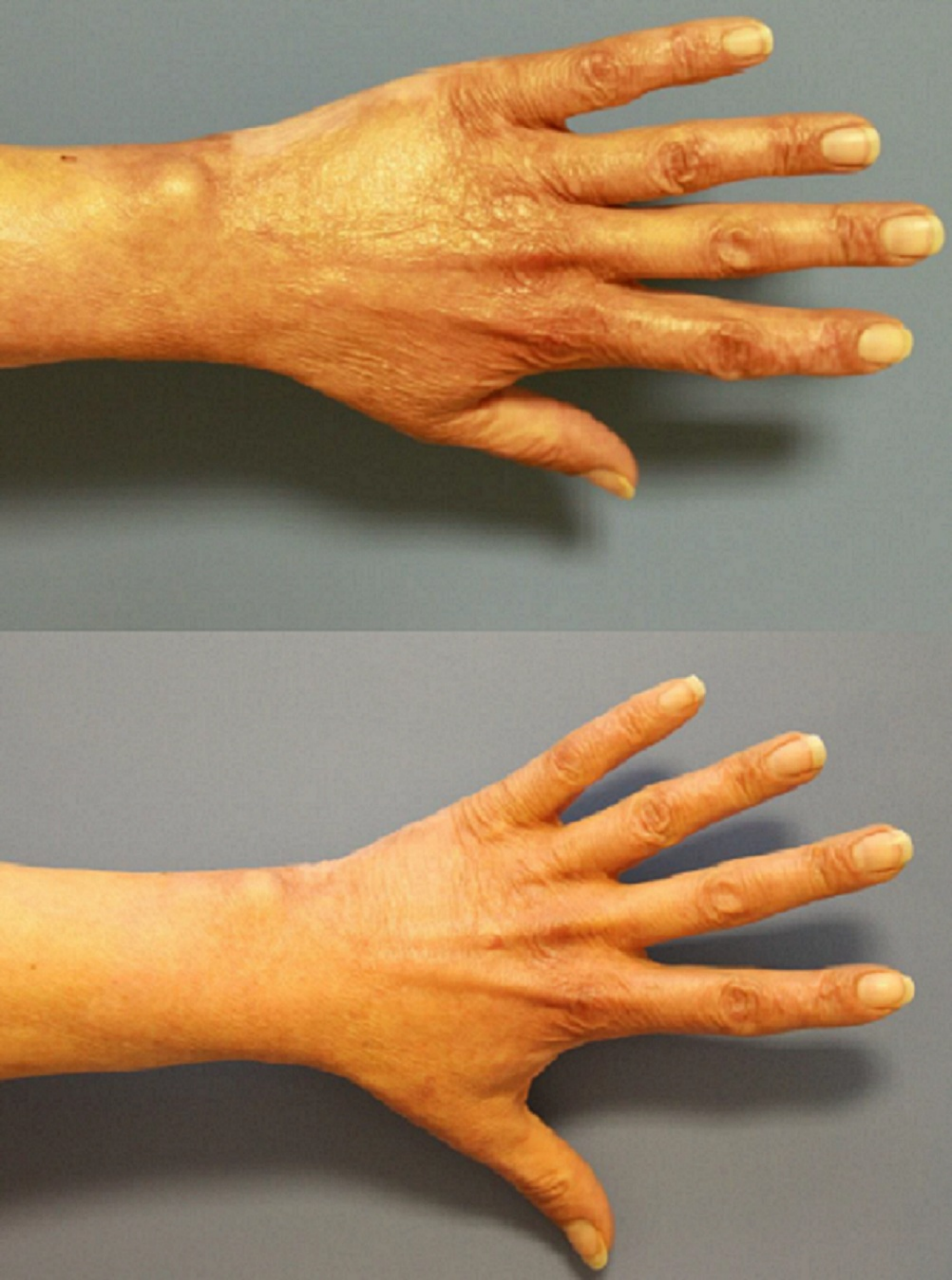
Patient 1, dorsal left hand, preoperatively (above) and one year postoperatively after needling (below)

**Figure 5:**
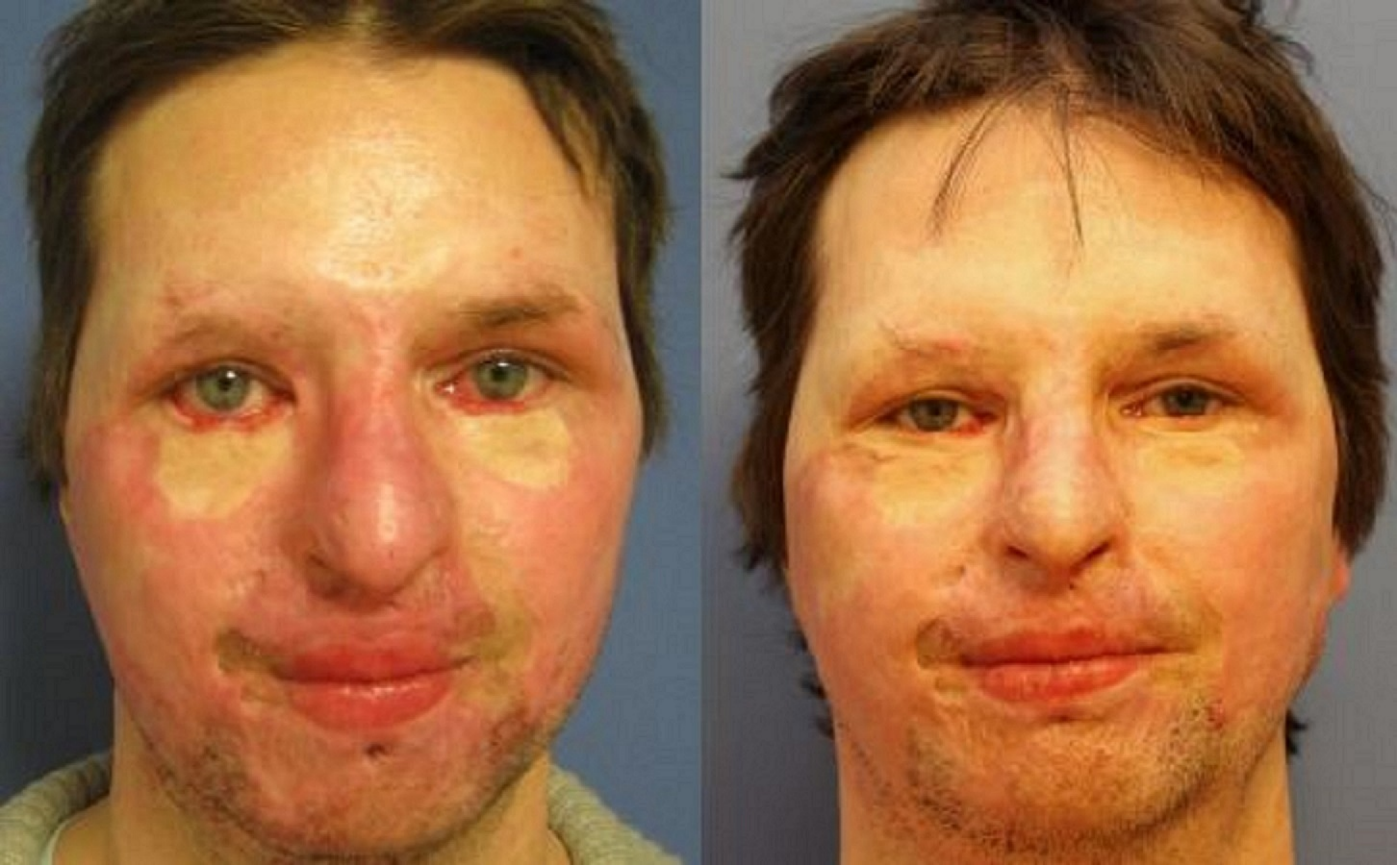
Patient 2, face frontal, preoperatively (left) and one year postoperatively after needling (right)

Hereafter, exemplary outcomes are shown (Figures 4-5).

Corneometer

**Figure 6:**
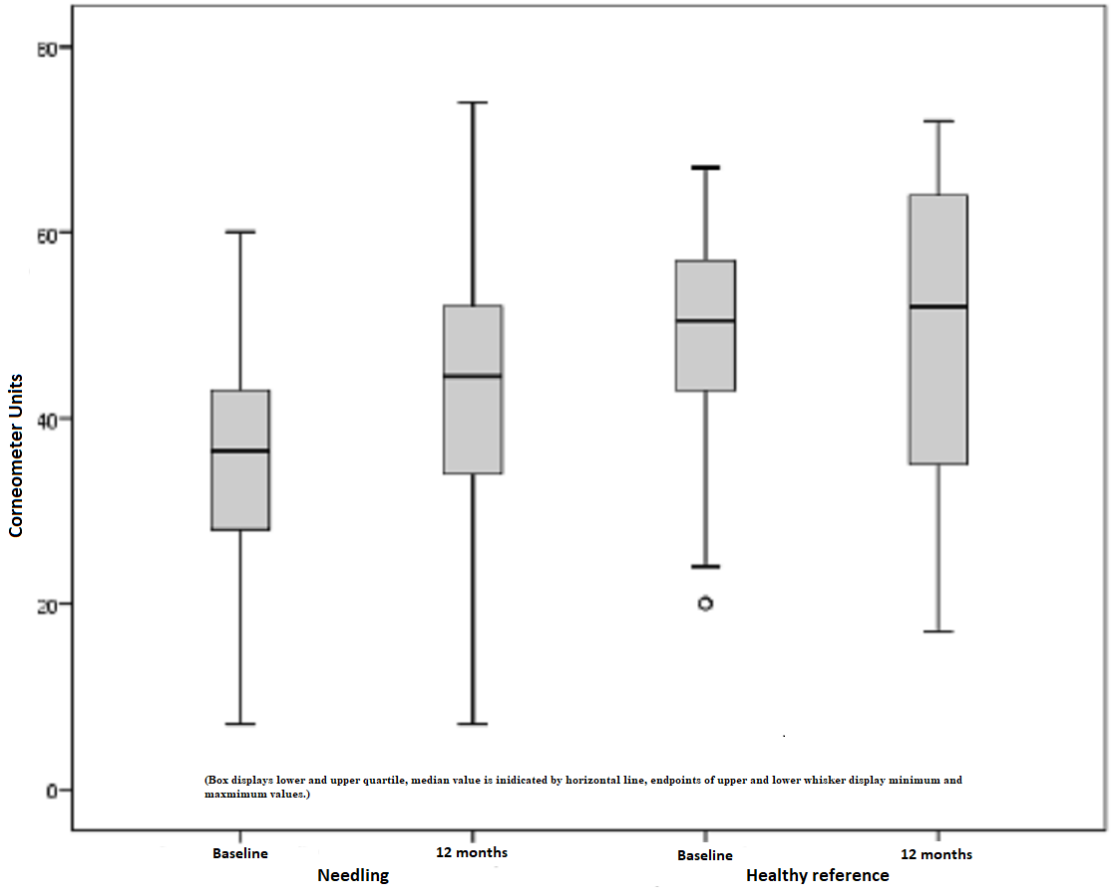
Moisture, pathological scars treated by medical needling and healthy skin preoperatively and one year later

**Figure 7:**
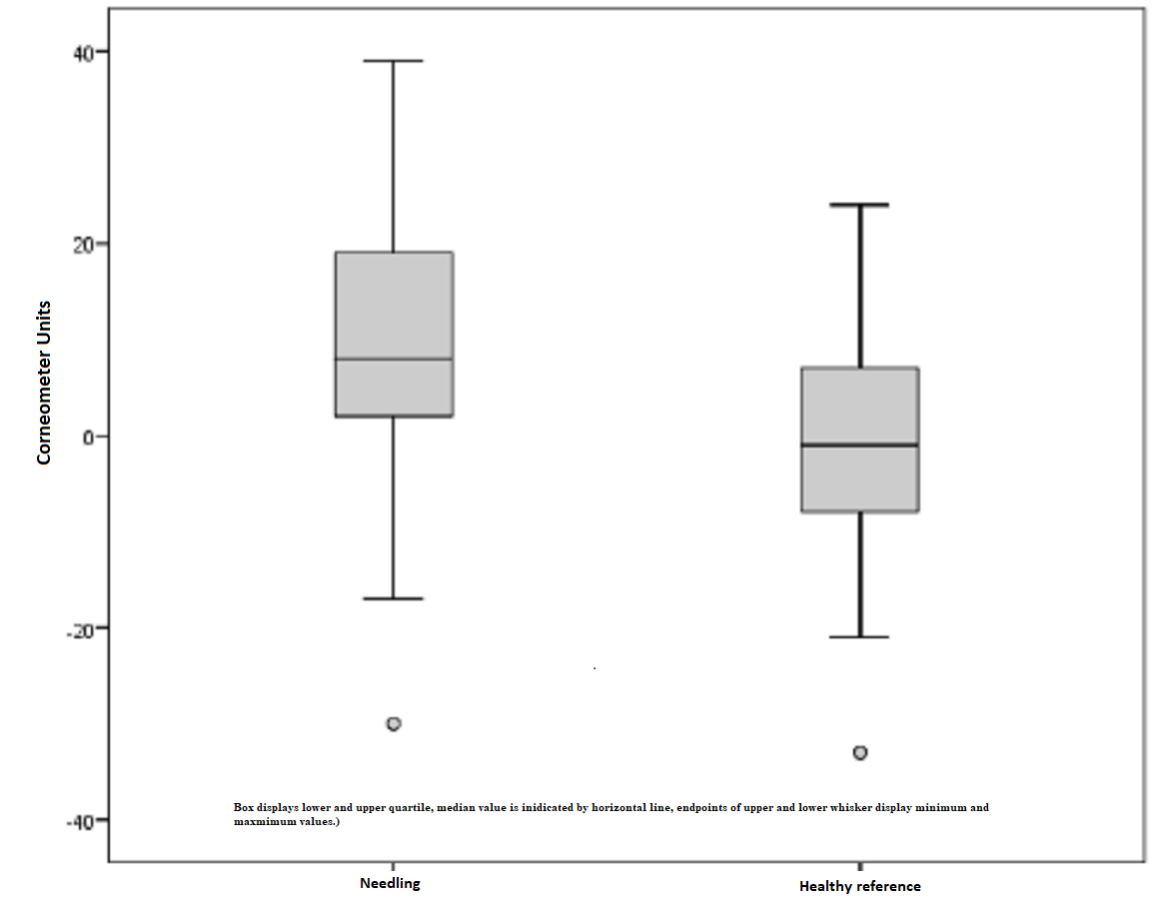
Difference in the moisture index for treated scars and healthy skin preoperatively and one year later

In the following figures, the results of the Corneometer measurements are depicted. Our subject collectively implied 38 scars out of 16 patients that revealed pathological values and, thus, were too dry. The pathological range is defined by a lower score compared to the standard values of healthy skin. Preoperatively, the mean moisture index was 34.79 points. An initial increase after six months continued to a mean of 43.76 points, which is an improvement of 26%. On the contrary, healthy skin showed a minimal reduction (1%) in the skin moisture, which is a non-significant change with p = 0.635 (Figure 6). Comparing the pre- and postoperative measurements of scars treated by medical needling, there was an average improvement of 8.87 points provided. The best effect was represented by a maximum of 38 points, an increase of 38 Corneometer unit points regarding moisture (Figure 7).

**Figure 8:**
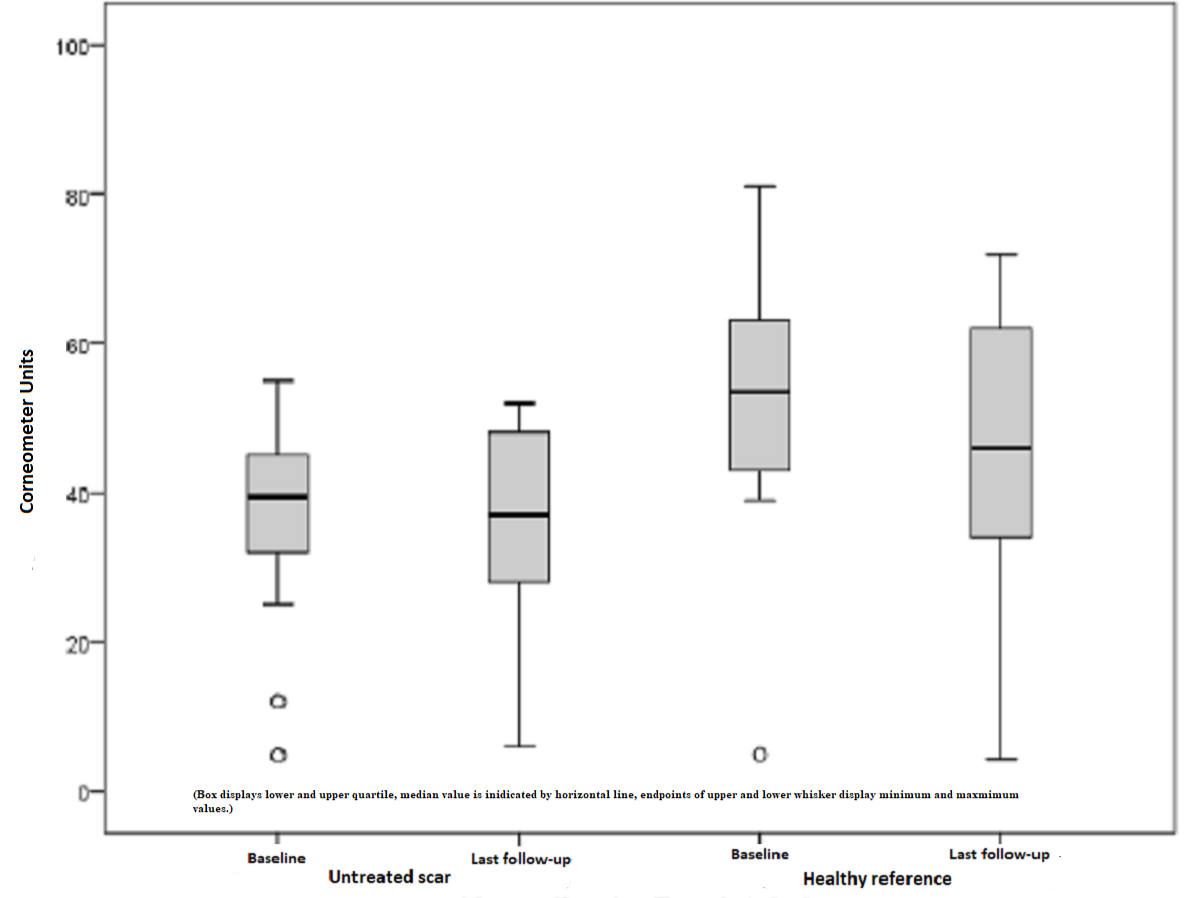
Moisture, untreated scars, and healthy skin preoperatively and one year later

The untreated scar was preoperatively measured with 38.31 points on average and postoperatively with 38.08. Hence, the scar situation remained more or less unchanged within the study period of one year and a statistical significance could not be recorded (p = 0.826). Once again, healthy skin measurements declared an 11% reduction in the skin moisture, which is not regarded as statistically significant with p = 0.148 (Figure 8).

**Figure 9:**
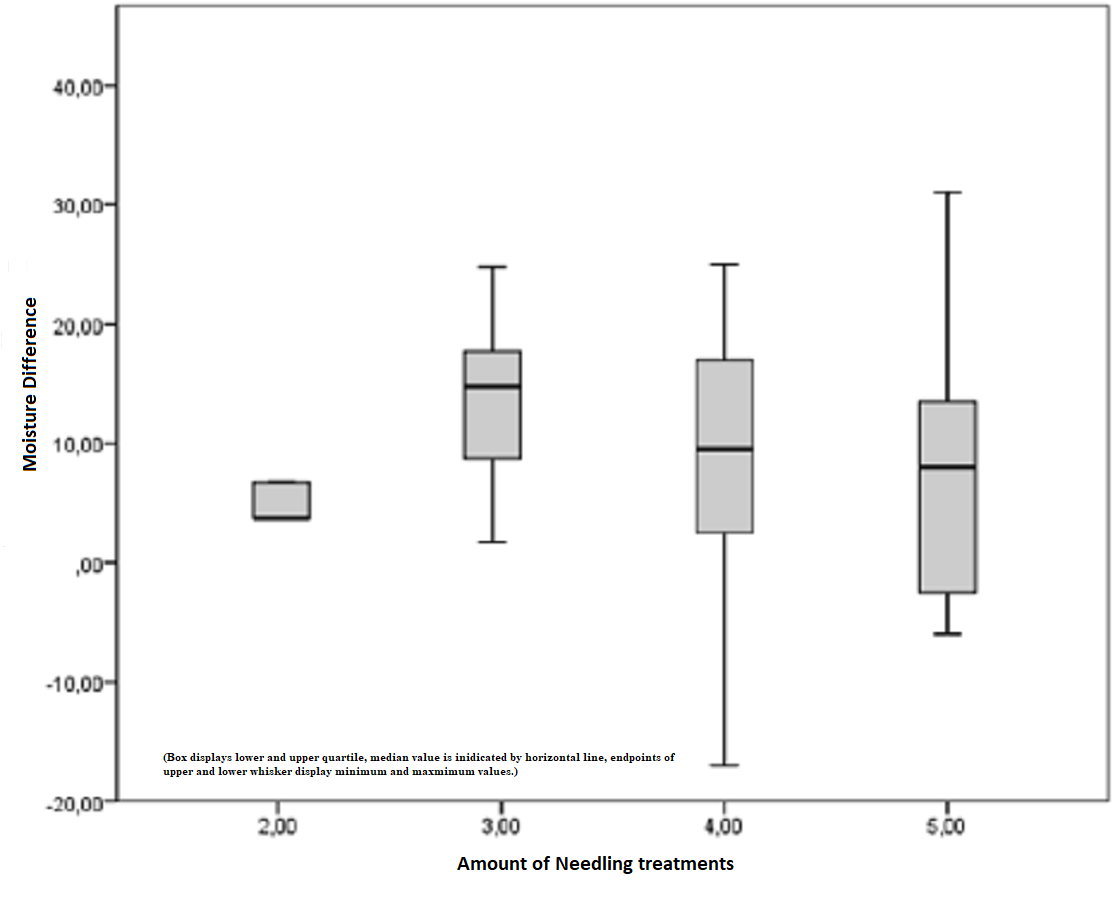
Difference between the pre- and postoperatively measured moisture index depending on the amount of treatment

Figure 9 shows the difference between the pre- and postoperatively measured moisture index in Corneometer units depending on the amount of treatment. The postoperative median for the moisture index after three treatments was 16 points, but it was half at the final examination, after five treatments. A significant improvement related to skin moisture is visible, whereas a proportional relation cannot be stated.

Tewameter

In the following figures, the results of the Tewameter measurements are depicted. TEWL can be two-dimensionally classified as pathologically increased or decreased. Both conditions were indications for medical needling and examined by using the Tewameter.

**Figure 10:**
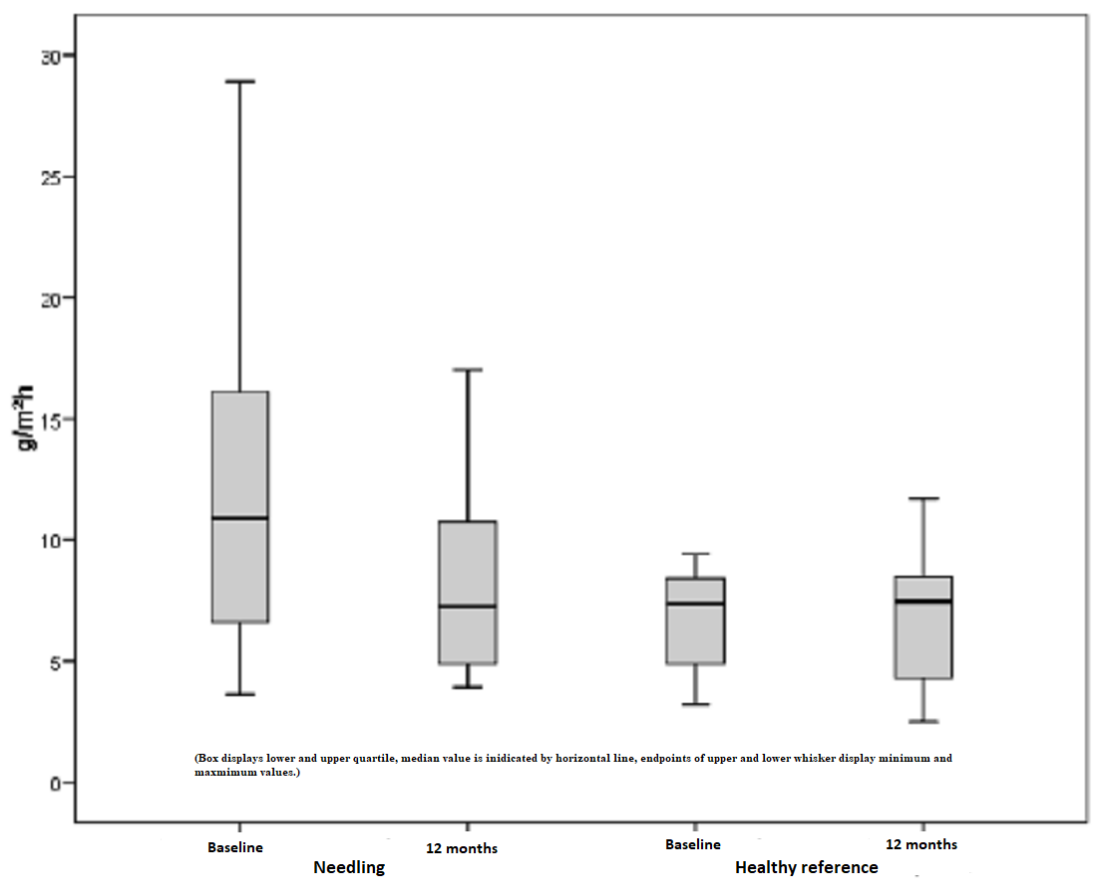
Increased TEWL, pathological scars treated by medical needling, and healthy skin preoperatively and one year later TEWL = Transepidermal water loss, increased values in scars treated by medical needling compared to healthy skin

**Figure 11:**
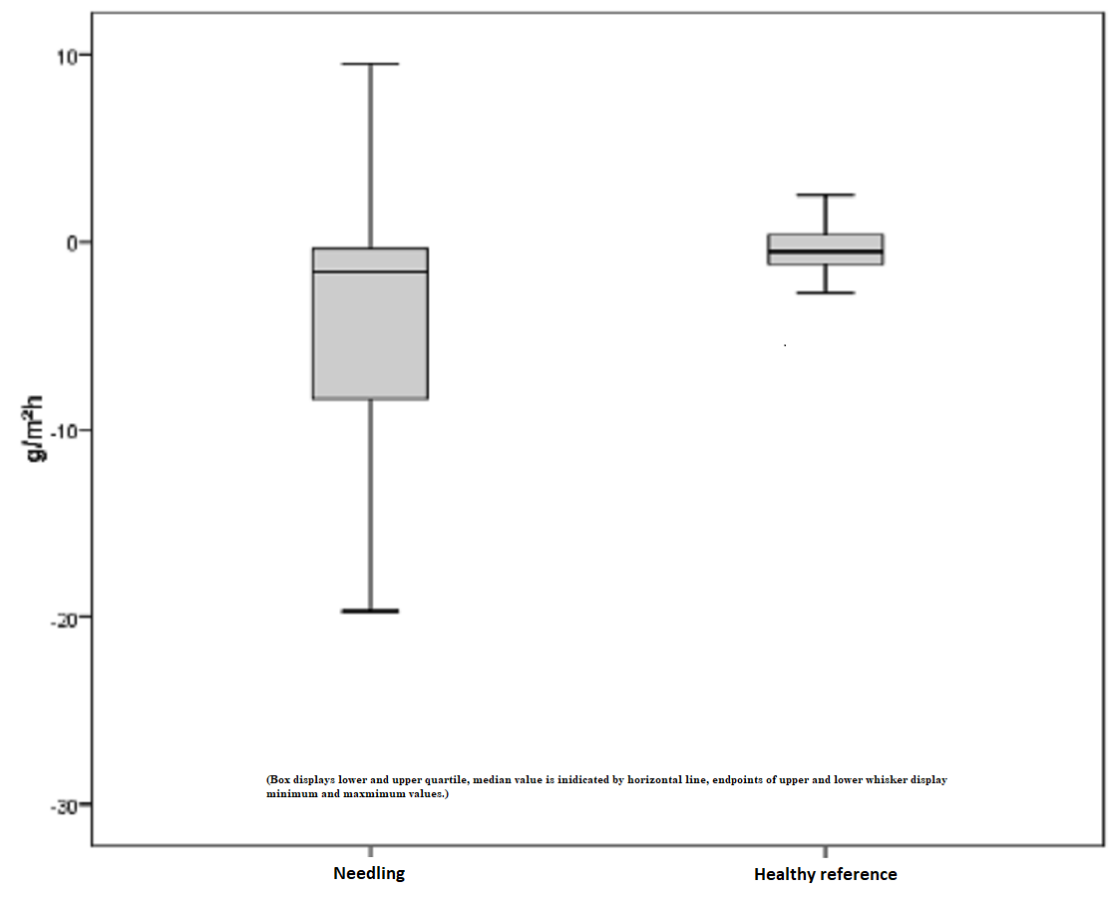
Difference of measured TEWL for pathologically increased scars and healthy skin preoperatively and one year later TEWL = Transepidermal water loss

A pathologically increased TEWL is defined by a higher value than the standard healthy reference. Our subject collectively implied 28 scars out of 13 patients, which revealed pathological values higher than the upper limit of a defined rage. Preoperatively, the mean TEWL index was 12.43 points. An initial decrease after six months continued to a mean TEWL index of 8.10 points after needling, which is an improvement of 35%. On the contrary, healthy skin showed a minimal reduction (1%) in TEWL, which is not statistically significant (Figure 10). Comparing the pre- and postoperative measurements of scars treated by medical needling, the mean improvement was 4.33 points. This result has a statistical significance, with p = 0.006. The best result was a TEWL index reduced by 19 Tewameter unit points (Figure 11).

**Figure 12:**
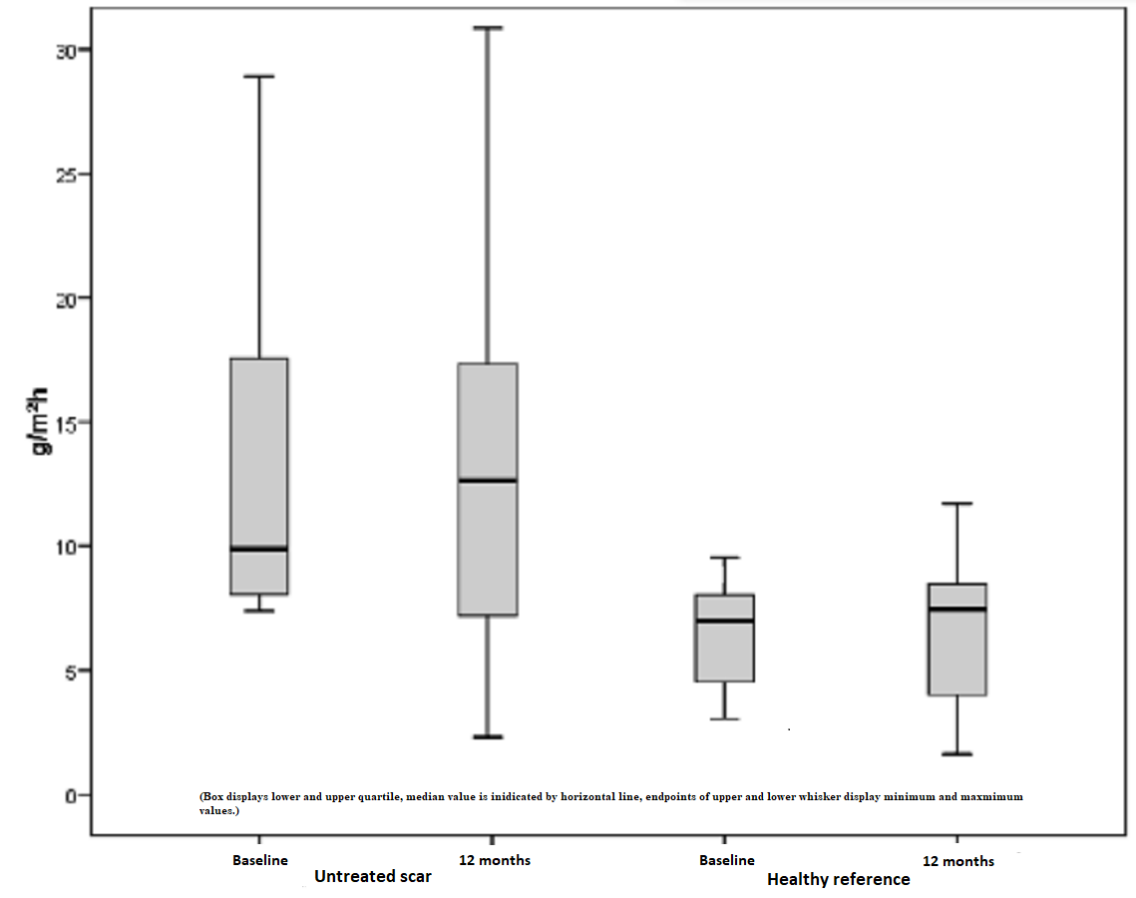
Increased TEWL, untreated scars, and healthy skin preoperatively and one year later TEWL = Transepidermal water loss, increased values of untreated scars compared to healthy skin

Less significant changes were evident when examining untreated scars and healthy skin. However, a decrease of 7% was recorded in untreated skin, with a mean TEWL index of 12.5 points preoperatively and 11.58 points postoperatively (Figure 12). Apart from that, a slight increase of 7% was notable, which is a renewed rise in the already increased TEWL. A statistical significance with p > 0.05 is not given.

**Figure 13:**
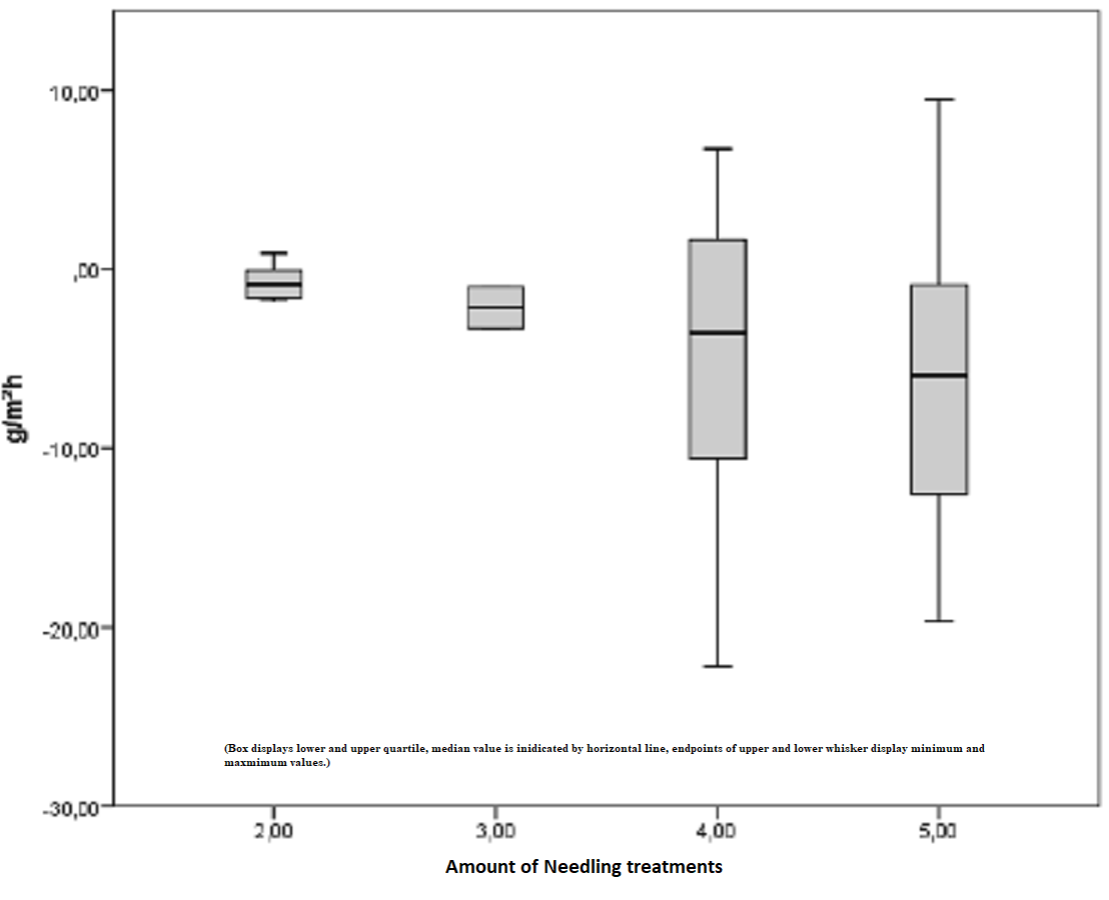
Increased TEWL, difference in the pre- and postoperatively measured TEWL index depending on the amount of treatment TEWL = Transepidermal water loss, improvements depending on the amount of treatment

Figure 13 shows the difference between the pre- and postoperatively measured TEWL index in Tewameter units depending on the amount of treatment. The postoperative median for the TEWL index after two treatments remained stable with -2.15 points, which changed after five treatments with a median of -5.95 points. Indeed, this result is trending toward an improvement in the pathological condition relative to the amount of treatment. However, linear regression in increased TEWL cannot be proved (p = 0.176).

**Figure 14:**
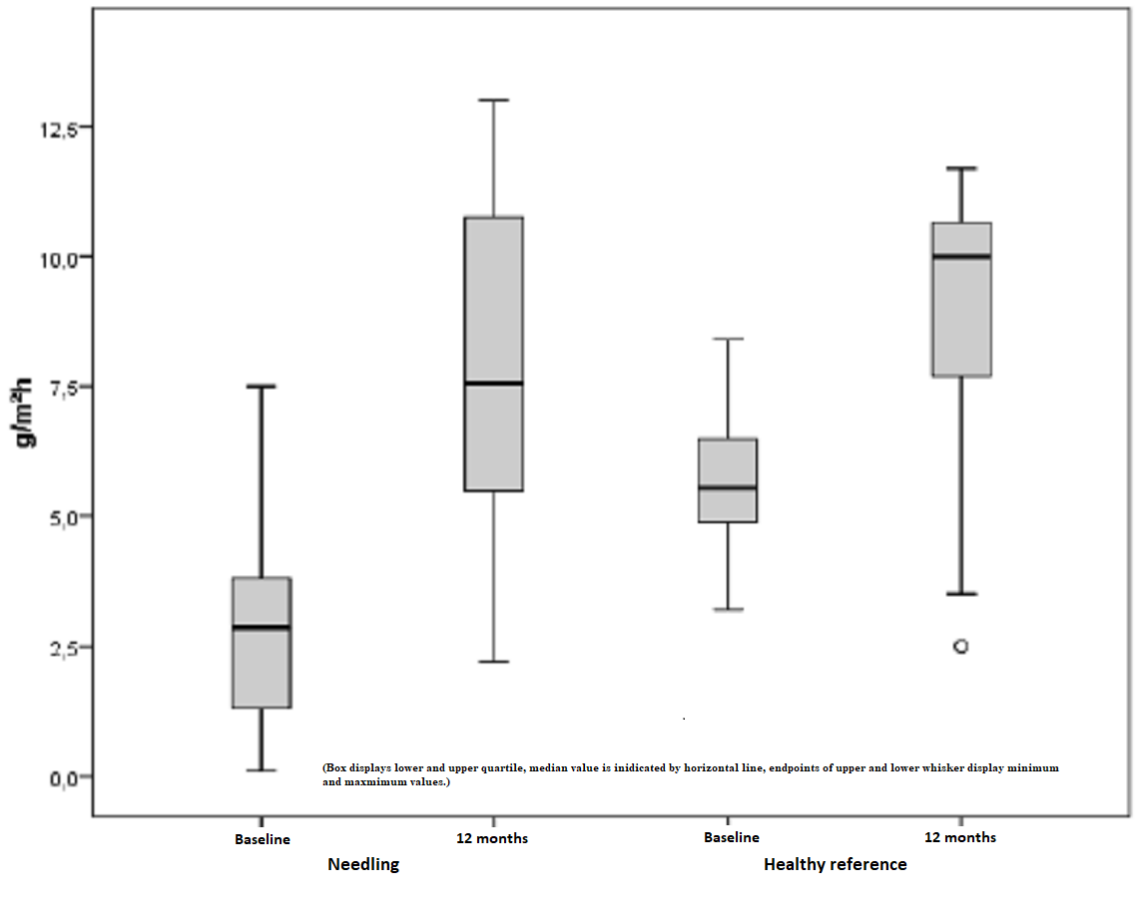
Decreased TEWL, pathological scars treated by medical needling, and healthy skin preoperatively and one year later TEWL = Transepidermal water loss, decreased values of scars treated by medical needling compared to healthy skin

A pathological value below the standard value of healthy skin applies to a pathologically decreased TEWL. In this study, 17 scars in of eight subjects were examined. Remarkable improvements showed a 140% increase in the TEWL index when considering a mean TEWL index of 3.15 points preoperatively and 7.58 points postoperatively. This outcome is statistically significant with p = 0.001 (Figure 14). Similiar to this, healthy skin achieved an improvement of approximately 50%, which is regarded as statistically significant with p = 0.004.

**Figure 15:**
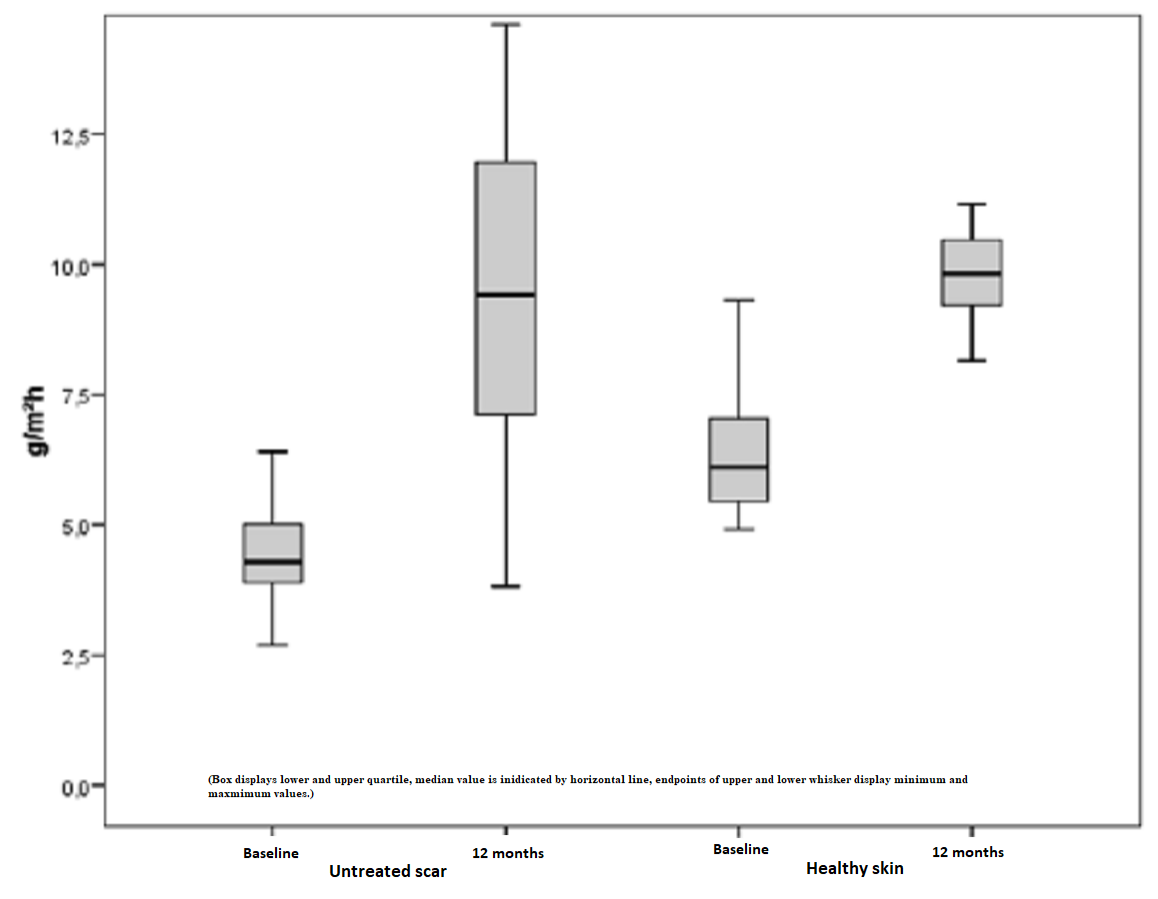
Decreased TEWL, untreated scars, and healthy skin preoperatively and one year later TEWL = Transepidermal water loss, decreased values of untreated scars compared to healthy skin

**Figure 16:**
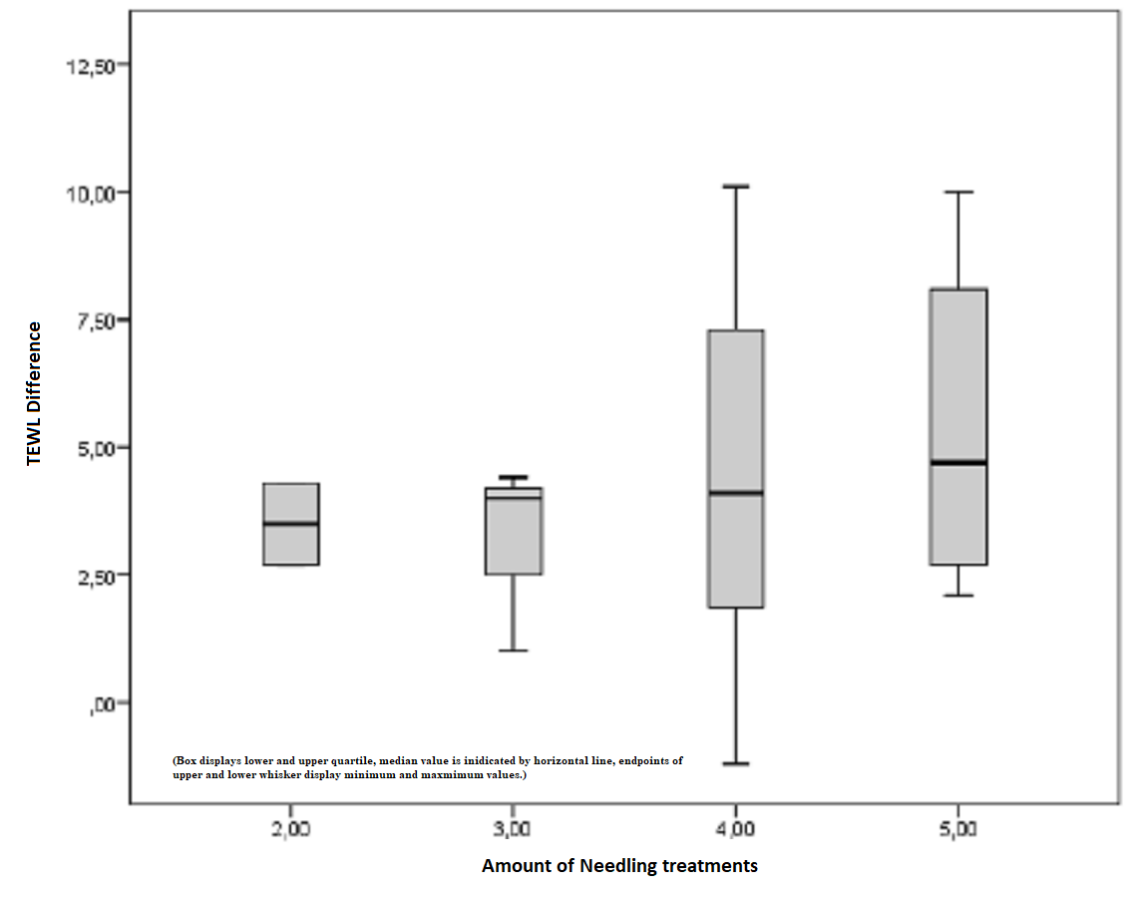
Difference between the pre- and postoperative measured TEWL index in Tewameter units depending on the amount of treatment TEWL = Transepidermal water loss, improvements depending on the amount of treatment

Furthermore, untreated scars showed similar values for both the median and the mean. The measured TEWL index within pre- and postoperative conditions is determined by an increase of 76%. However, this outcome is non-significant with p > 0.05 (Figure 15). Figure 16 depicts the difference between the pre- and postoperatively measured TEWL index in Tewameter units depending on the amount of treatment. The initial mean of 3.5 points increased to 6.38 points after the maximum of five treatments, which represents a positive trend toward an improvement due to repetitive needling.

## Discussion

There are various methods to treat mature hypertrophic burn scars. The quest for better scars has led to the establishment of not only non-invasive, conservative methods but also surgical options. Moderate success is often followed by several side effects and the risks of a potential degradation of the scar situation. Ablative methods, such as laser resurfacing or aggressive chemical treatments, are together defined by the same principle of the destruction of the skin structure, resulting in a complex post-interventional treatment [21]. Medical needling offers a controlled, simple, and quick method to improve the scar quality aesthetically and functionally. This ideal treatment overcomes the deficits of numerous methods available for treating scars such as ablative methods. It does not destroy epidermal structures but rather promotes the formation of physiological collagen instead of scar collagen. An ideal treatment induces and modulates the natural wound healing cascade after considering the expression of growth factors, such as TGF-ß, a tissue growth and transforming factor. Needling promotes the synthesis of dermal structures, such as collagen and elastin in terms of a dermal regeneration of the skin [22]. In this context, medical needling stimulates the gene expression of molecules, which are components of the extracellular matrix and responsible for its remodeling. Medical needling initiates the production of structural proteins, e.g. fibronectin and glycosaminoglycan molecules [23]. These proteins are capable of binding water and are responsible for skin hydration and moisture. Promoting the proliferation of skin cells, especially keratinocytes, changes the biophysical characteristics of the skin [24]. An increased liberation of keratinocytes causes an increase in epidermal thickness and measurable moisture. After apoptosis, dead keratinocytes build the stratum corneum. Multiple layers of corneocytes have a barrier function, which is crucial for maintaining homeostasis [25]. Cellular deficits of the epidermal function affect TEWL, which thus reflects the integrity of the epidermis [26]. Severe burns often cause the destruction of lipoprotein complexes in the stratum corneum, which impairs the barrier function and leads to pathological TEWL values. Pathologically increased or decreased TEWL values are equally possible and underly structural modifications of the epidermis. The consequences are dry and itchy scars with less flexibility and moisture due to increased TEWL. A decreased TEWL typically impairs the thermal regulation through evaporation. Medical needling ensures epidermal integrity in terms of regulating TEWL and reducing the risk of dehydration [27]. The study outcome showed remarkable improvements in skin roughness and rigidity, which both depend on the water content of the skin. The entire connective tissue framework appears thicker, denser, and smoother by inducing the post-needling regeneration process after treatment. All these parameters are interlinked with each other and need to be considered when it comes to evaluating moisture and related factors. With our study, we aimed at proving the effect of medical needling on scar quality with a special focus on skin moisture and TEWL. Objective and reliable data were supplied by measurements with the Corneometer and Tewameter. Scars treated by medical needling showed a measurable increase in skin moisture after six months and reached standard values in a physiological area after a year. One patient showed no increase in the moisture index postoperatively. Possible reasons might be an invalid or incorrect needling technique with low pressure or even an inadequate follow-up examination.

However, a non-responder-status is incorrect, as other parameters of the study were positively affected. Treated scars also indicate a decrease in TEWL. Not to be disregarded, TEWL is closely interlinked with epidermal moisture and is responsible for a measurable decrease in skin moisture as well. Improvements relative to the amount of therapy are evident, whereas a trend toward a proportional relation to an increase in skin moisture cannot be noticed. The patient cohort of this study is fairly limited to draw concrete conclusions. Examinations of both pathologically increased and decreased TEWL created a homogenous image. A statistical reduction of 35% was detected in the treatment of burn scars with pathologically increased values post-needling. Untreated scars of the same pathological condition achieved an improvement of 7%. A reduction of the mean is accompanied by an increase in the median at the same time. Considering the fact that the mean is more sensitive to external factors, such as emotional state (nervousness or stress), the median is more dominant in proving no significant improvement of untreated scars. Improvements in the TEWL index depending on the amount of treatment underline the thesis of an improved epidermal barrier function and thickness due to repetitive treatments with medical needling. In contrast, a pathologically decreased TEWL showed a remarkable improvement of 140% after treatment. At the same time, healthy skin with a pathologically decreased TEWL showed a significant increase of 52% as well. This gives rise to the assumption that an improvement of the status "decreased TEWL" of scars relative to the healthy reference cannot necessarily be associated with medical needling. The effect on hypertrophic burn scars with pathological TEWL below the standard range is of limited informative value. Added to that, the patient cohort is fairly limited to draw any concrete conclusions. POSAS contributed subjective and reliable data on the problem with itching and rigid scars. As medical needling influences the epidermal function and leads to a reduction in moisture stress in scars, the positive outcome of the subjectively rated pruritus can be considered a positive effect of medical needling. A controlled diffusion of water ensures a sufficient skin moisture, which consequently leads to less intense itching of hypertrophic scars. Significant improvements are manifested by reduced roughness and rigidity, which provides a great mobility of scars, especially in proximity to joints or other unfavorable areas. Since double-blind techniques were not employed in this study design, the patient's or observer's cognitive bias could have caused a subconscious influence on the evaluation of the scar quality.

## Conclusions

Our results show a subjective and objective improvement in skin moisture, TEWL, and scar surface. The established method of medical needling preserves the epidermis and improves the epidermal barrier function. Therefore, the liberation of several endogenous regenerating factors induces regenerative processes during the post-needling cascade, which improves the scarring in quality, function, and appearance. Furthermore, the needling treatment shows a significant impact on the skin‘s water balance by regulating moisturizing processes, such as the passive movement of water through the epidermis. Considering all examined parameters, medical needling is a promising approach for the treatment of mature hypertrophic burn scars.

## References

[1] Peck MD (2012). Epidemiology of burns throughout the world. Part II: Intentional burns in adults. Burns.

[2] Su CW, Alizadeh K, Boddie A, Lee RC (1998). The problem scar. Clin Plast Surg.

[3] Cheng B, Liu HW, Fu XB (2011). Update on pruritic mechanisms of hypertrophic scars in postburn patients: the potential role of opioids and their receptors. J Burn Care Res.

[4] Ya-Xian Z, Suetake T, Tagami H (1999). Number of cell layers of the stratum corneum in normal skin - relationship to the anatomical location on the body, age, sex and physical parameters. Arch Dermatol Res.

[5] Anthonissen M, Daly D, Peeters R (2015). Reliability of repeated measurements on post-burn scars with Corneometer CM 825®. Skin Res Technol.

[6] Meaume S, Le Pillouer-Prost A, Richert B, Roseeuw D, Vadoud J (2014). Management of scars updated practical guidelines and use of silicones. Eur J Dermatol.

[7] Busche MN, Roettger A, Herold C, Vogt PM, Rennekampff H-O (2016). Evaporative water loss in superficial to full thickness burns. Ann Plast Surg.

[8] Cohen S (1966). An investigation and fractional assessment of the evaporative water loss through normal skin and burn eschars using a microhygrometer. Plast Reconstr Surg.

[9] Gardien KL, Bass DC, de Vet HC, Middelkoop E (2016). Transepidermal water loss measured with the Tewameter TM300 in burn scars. Burns.

[10] Bleasdale B, Finnegan S, Murray K, Sean K, Steven PL (2015). The use of silicone adhesives for scar reduction. Adv Wound Care.

[11] Gilman TH (2003). Silicone sheet for treatment and prevention of hypertrophic scar: a new proposal for the mechanism of efficacy. Wound Repair Regen.

[12] Van den Kerckhove E, Stappaerts K, Boeckx W, Van den Hof B, Monstrey S, Van der Kelen A, De Cubber J (2001). Silicones in the rehabilitation of burns: a review and overview. Burns.

[13] Rabello FB, Souza CD, Farina Jr JA (2014). Update on hypertrophic scar treatment. Clinics.

[14] Baum TM, Busuito MJ (1998). Use of a glycerin-based gel sheeting in scar management. Adv Wound Care.

[15] Niessen FB, Spauwen PH, Schalkwijk J, Kon M (1999). On the nature of hypertrophic scars and keloids: a review. Plast Reconstr Surg.

[16] Manuskiatti W, Fitzpatrick RE (2002). Treatment response of keloidal and hypertrophic sternotomy scars: comparison among intralesional corticosteroid, 5-fluorouracil, and 585-nm flashlamp-pumped pulsed-dye laser treatments. Arch Dermatol.

[17] Hudson DA, Renshaw A (2006). An algorithm for the release of burn contractures of the extremities. Burns.

[18] Aust MC, Reimers K, Repenning C (2008). Percutaneous collagen induction: minimally invasive skin rejuvenation without risk of hyperpigmentation - fact or fiction?. Plast Reconstr Surg.

[19] Heinrich U, Koop U, Leneveu-Duchemin M-C (2003). Multicentre comparison of skin hydration in terms of physical-, physiological- and product-dependent parameters by the capacitive method (Corneometer CM 825). Int J Cosmet Sci.

[20] Aust MC, Des Fernandes, Kolokythas P, Kaplan HM, Vogt PM (2008). Percutaneous collagen induction therapy: an alternative treatment for scars, wrinkles, and skin laxity. Plast Reconstr Surg.

[21] Anzarut A, Olson J, Singh P, Rowe BH, Tredget EE (2009). The effectiveness of pressure garment therapy for the prevention of abnormal scarring after burn injury: a meta-analysis. J Plast Reconstr Aesthet.

[22] Ferguson MWJ, O'Kane S (2004). Scar-free healing: From embryonic mechanisms to adult therapeutic intervention. Philos Trans R Soc Lond B Biol Sci.

[23] Aust MC, Reimers K, Gohritz A (2008). Percutaneous collagen induction. Scarless skin rejuvenation: fact or fiction?. Clin Exp.

[24] Chapellier B, Mark M, Messaddeq N (2002). Physiological and retinoid-induced proliferations of epidermis basal keratinocytes are differently controlled. EMBO J.

[25] Tagami H (2008). Location-related differences in structure and function of the stratum corneum with special emphasis on those of the facial skin. Int J Cosmet Sci.

[26] Gfesser M, Rakoski J, Ring J (1996). The disturbance of epidermal barrier function in atopy patch test reactions in atopic eczema. Br J Dermatol.

[27] Berman B, Viera MH, Amini S, Huo R, Jones IS (2008). Prevention and management of hypertrophic scars and keloids after burns in children. J Craniofac Surg.

